# Gender difference in appendicular muscle strength: determinant of the quality of life in the older Taiwanese

**DOI:** 10.18632/aging.204297

**Published:** 2022-09-19

**Authors:** Mei-Jung Chen, Pi-Shao Ko, Meng-Chang Lee, Sui-Lung Su, Shu Yu

**Affiliations:** 1 School of Nursing, National Yang Ming Chiao Tung University, Taipei 112304, Taiwan; 2 Department of Nursing, Tri-Service General Hospital Songshan Branch, Taipei 105309, Taiwan; 3 School of Public Health, National Defense Medical Center, Taipei 114201, Taiwan

**Keywords:** quality of life (QoL), muscle mass percentage, fat mass percentage, 12-Item Short Form Survey (SF-12), gender difference

## Abstract

Background: The loss of skeletal muscle mass by aging determines the health status and the quality of life (QoL).

Objective: To examine the relationships between appendicular muscle strength and the QoL of elderly adults in gender difference.

Methods: This was a cross-sectional study, in which 690 subjects who participated in older adults health examination in the health management center of Tri-Service General Hospital from 2018 to 2021. A structured questionnaire was used to collect basic demographic data. The 12-Item Short Form Survey (SF-12) was used to evaluate the QoL of subjects. Their grip strength and gait speed were measured, and Dual-energy X-ray absorptiometry was used to measure muscle mass and other body composition data. Multivariate regression analysis was used to examine the relationships between upper and lower limb muscle strength and the QoL of older adults.

Results: In men, legs muscle mass percentage (LegsMM%) (β = 3.67; 95% CI: 0.64–6.69; *p* = 0.018) and gait speed (β = 6.09; 95% CI: 3.88–8.30; *p* < 0.001) were positively associated with physical component summary (PCS) scores, and gait speed (β = 4.63; 95% CI: 2.66–6.60; *p* < 0.001) was also related to an improvement mental component summary (MCS) scores. In women, arms muscle mass percentage (ArmsMM%) (β = 6.50; 95% CI: 2.34–10.66; *p* = 0.002) and grip strength (β = 10.54; 95% CI: 6.27–14.81; *p* < 0.001) had the greatest effect on improving PCS scores, whereas grip strength (β = 7.58; 95% CI 4.00–11.17; *p* < 0.001) was also found to help improve MCS scores.

Conclusions: Men should focus on lower limb training, whereas females should focus on upper limb training to effectively improve their QoL. Appropriate exercise interventions should be designed for different genders for the promotion of the healthy aging policy.

## INTRODUCTION

Aging is an important issue in almost all developed countries. The global population of elderly individuals aged ≥65 years is expected to increase 28.9% from 771 million to 994 million from 2022 to 2030 [1]. Currently, public health policy majorly focused on the improvement of elderly quality of life (QoL) [2].

Aging plays a pivotal role in the modification of body composition. Generally, skeletal muscle mass decreases and fat increases in the older adults [3]. In older adults, changes in body composition are often associated with insufficient exercise, malnutrition, disability, obesity, and cardiovascular diseases [4]. Among these changes, skeletal muscles play an important role in physical function maintenance, muscle mass decreases by 3–8% every 10 years after coming to apex at 30 years old, and this process deteriorates after 50 years old [5–7]. However, physical exercise averts detrimental process caused by aging in maintenance of skeletal muscles or physical function [8, 9].

Gender determines the body composition; men have a higher ratio of muscle to fat, contrary to women [10, 11]. Age compounds gender-dependent body composition, plummeting muscle mass in men, a case in point [12]. Compared to loss of muscle mass in men, decrease of muscle strength carries more weighing values in women [12–16].

QoL is a complex concept that includes individual objective factors, subjective factors, and sociocultural effects [17]. The QoL of elderly individuals can be assessed based on physical functional status and ability to independently complete activities of daily living [18]. In addition to worsening health due to chronic diseases that occur with age, decrease in physical function is also common in elderly individuals, thereby affecting health status and QoL [19]. Therefore, the relationship between age-related changes (such as body composition) and QoL have been widely researched [20–22]. Among the changes that may lead to negative effects on QoL, more attention should be paid to reduction in muscle mass and strength and increase in fat mass.

Decreased muscle mass and strength are important indicators for sarcopenia and dynapenia. Sarcopenia refers to decreased muscle mass, function, and strength and has adverse effects on health in elderly individuals, thereby affecting QoL [13, 23, 24]. On the other hand, dynapenia refers to age-related loss in muscle strength and presents as decreased muscle strength. This limits the mobility of elderly individuals, affects their QoL, and increases the risk of death. In addition, dynapenia occurs earlier than sarcopenia and is an important prognostic indicator of disability in elderly individuals [24–27]. It can be seen that muscle mass, muscle strength, and physical function are associated with the health and QoL of elderly individuals. A study pointed out that upper limb muscle strength plays an important role in many activities of daily living and may affect individual independence and QoL [28]. Handgrip strength (HGS) measurement is considered to be a good indicator of overall muscle strength and function in elderly individuals [29, 30]. However, other studies pointed out that lower limb muscle strength is associated with QoL as lower limb muscle strength is a factor that determines elderly individuals’ level of independence in terms of daily living [20]. Although the correlation between muscle strength and QoL of elderly individuals has been extensively studied, there are gender differences in body composition with aging and a lack of studies that systematically examine the correlation between body composition and the QoL of elderly individuals. Therefore, this study aimed to examine the relationship between upper and lower limb muscle strength and the QoL of elderly individuals and determine the presence in gender difference.

## MATERIALS AND METHODS

### Study participants

This study used a cross-sectional research design, and elderly individuals who were undergoing health examinations at Tri-Service General Hospital were recruited between March 2018 and October 2021. All the participants did not consume alcohol for at least 48 h, perform vigorous exercise for at least 12 h, or fasting for at least 12 h prior to the examination. Structured questionnaires were used to obtain basic demographic data from the study subjects. A total of 690 elderly individuals (297 males and 393 females) were included in the study.

### Anthropometric measurements

Body weight was measured by a standard scale as subjects wore light clothing and no shoes; barefoot standing height was measured by a wall-mounted stadiometer; and body mass index (BMI) was calculated using the following formula: weight in kilograms/(height in meters)^2^.

### Body composition measurements

Dual-energy X-ray absorptiometry (DXA) was used for measuring body composition, including fat and muscle masses. The participants were dressed in cotton robes without metal attachments and were placed in a supine position in the center of the scanning field with their palms facing downwards; arms lying away from their body, either straight or at a slight angle; feet in a neutral position; and face facing upwards with their chin in a neutral position. The scan took approximately 180 s to complete, and the dose of radiation per individual was 0.01 mGy (1.0 mrad). Muscle and fat mass percentage were calculated as follows: muscle mass percentage (MM%) = muscle mass/weight, appendicular muscle mass percentage (AMM%) = appendicular muscle mass/weight, arms muscle mass percentage (ArmsMM%) = arms muscle mass/weight, legs muscle mass percentage (LegsMM%) = legs muscle mass/weight, trunk muscle mass percentage (TrunkMM%) = trunk muscle mass/weight, fat mass percentage (FM%) = fat mass/weight, appendicular fat mass percentage (AFM%) = appendicular fat mass/weight, arms fat mass percentage (ArmsFM%) = arms fat mass/weight, legs fat mass percentage (LegsFM%) = legs fat mass/weight, and trunk fat mass percentage (TrunkFM%) = trunk fat mass/weight. The fat-to-muscle ratio (FMR) was calculated as follows: fat mass/muscle mass.

### Muscle strength measurements

#### HGS

In the present study, HGS was used as an indicator of upper limb muscle strength and was assessed by using a Hydraulic Hand Dynamometer (JAMAR Plus+). Evaluation of grip strength was complied with standard principles that mentioned in several papers [31–33]; participants were asked to stand and used only the dominant hand with the elbow joint at 90°C and shoulder joint at 180°C of flexion. Additionally, the wrist and trunk were at neutral positions. Participants had their HGS measured three times and the best of the three attempts was recorded.

#### Gait speed

In the present study, gait speed was used as an indicator of lower limb muscle strength, and a 15-feet (4.572 m) unobstructed flat ground was used for gait speed testing. Two end lines were taped on the ground; one at the beginning and one at the end of the 15-feet ground. Participants were asked to walk as quickly and safely as possible, and a stopwatch was used to record the time.

### QoL measurements

The 12-Item Short Form Health Survey (SF-12) was used to assess each participant’s QoL. It consists of 12 questions covering 8 health domains: physical functioning (PF), social functioning (SF), role-physical (RP), role-emotional (RE), mental health (ME), energy/vitality (VT), bodily pain (BP) and general health perception (GH) [31, 32]. The questions were combined, scored, and weighted to create the physical component summary (PCS) and mental component summary (MCS) scores ranging from 0 (lowest level of health) to 100 (highest level of health). The PCS scores comprised of PF, RP, BP, and GH domains, and the MCS scores comprised of SF, RE, ME, and VT domains.

### Statistical analysis

Categorical and continuous variables were presented as a number (proportion) and mean ± standard deviation. We used Student *t*-test and χ^2^ test (with Fisher’s exact test when appropriate) to compare the demographic data, anthropometric data, body composition, SF-12 domains, HGS and gait speed between male and female participants. Linear regression was used to test the effects of each independent variable on each dependent variable. To determine the independent association of body composition and muscle strength, we used multiple regression analysis with adjustment for selected demographic and anthropometric variables. Statistical analyses were performed using SPSS 22.0 (SPSS Inc., Chicago, IL, USA) and R 3.5.2 (R Project for Statistical Computing, Vienna, Austria). A *p*-value of <0.05 was considered as the level of statistical significance.

## RESULTS

### Population characteristics

The basic clinical characteristics of the study population are summarized in Table 1. The participants in this study were aged 72.21 ± 6.88 years (male: 72.97 ± 7.73, female: 71.64 ± 6.11, *p* = 0.012). Males had significantly higher BMI and higher components of body composition, such as muscle mass than those in females, whereas fat mass were significantly higher in females exception for trunk fat mass. Males had higher SF-12 scores than females. In addition, males had significantly higher HGS and gait speed than those in females.

**Table 1:** Basic demographic and clinical data.

	**Total (*n* = 690)**	**Male (*n* = 297)**	**Female (*n* = 393)**	***p*-value**
Demography				
Age	72.21 ± 6.88	72.97 ± 7.73	71.64 ± 6.11	0.012^*^
Marital status				
Unmarried/Widow/Divorced	147 (21.8%)	21 (7.3%)	126 (32.8%)	<0.001^*^
Married/Cohabitant	526 (78.2%)	268 (92.7%)	258 (67.2%)
Smoke				
None	585 (86.9%)	207 (71.6%)	378 (98.4%)	<0.001^*^
Current/Former	88 (13.1%)	82 (28.4%)	6 (1.6%)
Alcohol consumption				
None	648 (96.3%)	267 (92.4%)	381 (99.2%)	<0.001^*^
>20 g per day	25 (3.7%)	22 (7.6%)	3 (0.8%)
Anthropometry				
Height	158.98 ± 7.97	165.33 ± 6.02	154.18 ± 5.53	<0.001^*^
Weight	61.71 ± 10.97	68.33 ± 9.91	56.70 ± 8.88	<0.001^*^
Body Mass Index	24.28 ± 3.50	24.95 ± 3.08	23.78 ± 3.71	<0.001^*^
Body composition				
Muscle mass	39.64 ± 7.96	47.31 ± 5.24	33.85 ± 3.52	<0.001^*^
Appendicular muscle mass	16.60 ± 3.90	20.32 ± 2.65	13.78 ± 1.73	<0.001^*^
Arms muscle mass	4.25 ± 1.16	5.35 ± 0.81	3.41 ± 0.52	<0.001^*^
Legs muscle mass	12.35 ± 2.79	14.97 ± 1.96	10.37 ± 1.30	<0.001^*^
Trunk muscle mass	19.44 ± 3.77	22.91 ± 2.64	16.81 ± 1.91	<0.001^*^
Fat mass	19.44 ± 6.25	18.10 ± 5.89	20.46 ± 6.33	<0.001^*^
Appendicular fat mass	7.14 ± 2.65	5.91 ± 1.97	8.08 ± 2.72	<0.001^*^
Arms fat mass	1.78 ± 0.74	1.46 ± 0.55	2.02 ± 0.77	<0.001^*^
Legs fat mass	5.36 ± 2.04	4.45 ± 1.50	6.06 ± 2.12	<0.001^*^
Trunk fat mass	11.49 ± 3.88	11.44 ± 4.00	11.53 ± 3.79	0.765
Fat-to-muscle ratio	0.51 ± 0.18	0.38 ± 0.11	0.60 ± 0.17	<0.001^*^
SF-12				
Physical component summary scores	68.26 ± 22.59	73.33 ± 20.05	64.45 ± 23.65	<0.001^*^
Physical functioning	77.00 ± 28.87	84.08 ± 24.06	71.69 ± 30.99	<0.001^*^
Role-physical	68.92 ± 43.29	75.61 ± 40.20	63.90 ± 44.87	<0.001^*^
Bodily pain	87.76 ± 20.41	91.00 ± 16.83	85.32 ± 22.44	<0.001^*^
General health	39.35 ± 24.05	42.65 ± 24.03	36.88 ± 23.80	0.002^*^
Mental component summary scores	78.32 ± 19.49	81.24 ± 18.23	76.13 ± 20.13	0.001^*^
Vitality	68.04 ± 28.01	70.66 ± 27.44	66.08 ± 28.31	0.036^*^
Social functioning	86.20 ± 22.20	87.80 ± 22.16	85.00 ± 22.19	0.105
Role-emotional	77.23 ± 37.03	82.01 ± 33.15	73.64 ± 39.36	0.004^*^
Mental health	81.82 ± 19.18	84.50 ± 16.58	79.82 ± 20.72	0.002^*^
Handgrip strength	27.72 ± 10.09	35.89 ± 8.64	21.55 ± 5.82	<0.001^*^
Gait speed	1.10 ± 0.33	1.13 ± 0.35	1.07 ± 0.32	0.024^*^

^*^p < 0.05.

### Linear regression model of demographic and anthropometric data with QoL

In Table 2, simple linear regression analysis identified the relevant variables associated with the SF-12 scores. PCS scores were negatively associated with females (*p* < 0.001) and age (*p* = 0.010), and positively associated with current or former smoking (*p* = 0.011). MCS scores were negatively associated with females (*p* = 0.001) and BMI (*p* = 0.019) and positively associated with married/cohabitant marital status (*p* = 0.015).

**Table 2:** Linear regression model of demographic and anthropometric data with QoL.

	**PCS**			**MCS**		
	**β (95% CI)**		***p*-value**	**β (95% CI)**		***p*-value**
Demography						
Sex			<0.001^*^			0.001^*^
Male	Ref.			Ref.		
Female	−8.89	(−12.27~−5.50)		−5.11	(−8.06~−2.16)	
Age	−0.33	(−0.58~−0.08)	0.010^*^	−0.06	(−0.28~0.15)	0.569
Marital status			0.093			0.015^*^
Unmarried/Widow/Divorced	Ref.			Ref.		
Married/Cohabitant	3.54	(−0.59~7.67)	0.093	4.39	(0.84~7.94)	
Smoking status			0.011^*^			0.408
None	Ref.			Ref.		
Current/former	6.58	(1.54~11.62)		1.84	(−2.53~6.22)	
Alcohol consumption			0.371			0.104
None	Ref.			Ref.		
>20g per day	4.12	(−4.91~13.15)		6.45	(−1.33~14.23)	
Anthropometry						
Height	0.50	(0.29~0.71)	<0.001^*^	0.19	(0.01~0.38)	0.040^*^
Weight	0.14	(−0.02~0.29)	0.091	−0.02	(-0.16~0.11)	0.733
BMI	−0.42	(−0.92~0.07)	0.096	−0.51	(−0.94~−0.09)	0.019^*^

^*^p < 0.05; Abbreviations: CI: Confidence interval; PCS: Physical component summary scores; MCS: Mental component summary scores.

### Linear regression model of body compositions and muscle strength with QoL

The body composition and muscle strength factors associated with the SF-12 PCS and MCS scores are shown in Table 3. PCS scores, after adjusting for sex, age, height, and smoking status, were positively associated with muscle mass percentage and muscle strength, exception for TrunkMM% (*p* = 0.206), but negatively associated with fat mass and FMR. MCS scores, after adjusting for sex, height, and marital status, were positively associated with muscle mass and muscle strength, exception for ArmsMM% (*p* = 0.065) and TrunkMM% (*p* = 0.170), but negatively associated with fat mass and FMR, exception for AFM% (*p* = 0.052) and LegsFM% (*p* = 0.216).

**Table 3:** The correlation of body compositions and muscle strength with QoL by linear regression model.

	**PCS**				**MCS**			
	**Standardized β (95% CI)**	***p*-value**	**Standardized Adj-β (95% CI)^a^**	***p*-value**	**Standardized β (95% CI)**	***p*-value**	**Standardized Adj-β (95% CI)^b^**	***p*-value**
Body composition								
MM%	4.68 (2.98~6.38)	<0.001^*^	2.77 (0.69~4.85)	0.009^*^	2.96 (1.48~4.44)	<0.001^*^	2.14 (0.33~3.95)	0.021^*^
AMM%	5.97 (4.29~7.65)	<0.001^*^	4.65 (2.36~6.94)	<0.001^*^	3.59 (2.12~5.06)	<0.001^*^	3.21 (1.23~5.18)	0.002^*^
ArmsMM%	5.41 (3.73~7.09)	<0.001^*^	3.58 (1.03~6.13)	0.006^*^	2.95 (1.47~4.42)	<0.001^*^	2.05 (−0.12~4.23)	0.065
LegsMM%	5.86 (4.18~7.54)	<0.001^*^	4.40 (2.27~6.52)	<0.001^*^	3.66 (2.19~5.13)	<0.001^*^	3.18 (1.34~5.01)	0.001^*^
TrunkMM%	3.20 (1.49~4.92)	<0.001^*^	1.25 (−0.68~3.18)	0.206	2.15 (0.66~3.63)	0.005^*^	1.19 (−0.51~2.88)	0.170
FM%	−5.04 (−6.73~−3.34)	<0.001^*^	−3.19 (−5.30~−1.08)	0.003^*^	−2.98 (−4.46~−1.50)	<0.001^*^	−2.16 (−4.02~−0.31)	0.022^*^
AFM%	−5.44 (−7.15~−3.74)	<0.001^*^	−3.93 (−6.36~−1.51)	0.002^*^	−2.95 (−4.45~−1.46)	<0.001^*^	−2.12 (−4.25~0.02)	0.052
ArmsFM%	−5.24 (−6.95~−3.53)	<0.001^*^	−3.40 (−5.65~−1.16)	0.003^*^	−3.51 (−4.99~−2.02)	<0.001^*^	−3.11 (−5.08~−1.15)	0.002^*^
LegsFM%	−5.09 (−6.81~−3.38)	<0.001^*^	−3.29 (−5.61~−0.97)	0.006^*^	−2.53 (−4.03~−1.02)	0.001^*^	−1.29 (−3.34~0.75)	0.216
TrunkFM%	−3.77 (−5.49~−2.05)	<0.001^*^	−2.10 (−3.94~−0.26)	0.026^*^	−2.47 (−3.96~−0.98)	0.001^*^	−1.70 (−3.31~−0.08)	0.040^*^
FMR	−5.53 (−0.21~−3.85)	<0.001^*^	−4.03 (−6.13 ~ 1.93)	<0.001^*^	−3.40 (−4.87~−1.93)	<0.001^*^	−2.92 (−4.76~−1.08)	0.002^*^
Muscle strength								
Handgrip strength	6.27 (4.62~7.92)	<0.001^*^	5.33 (2.75~7.91)	<0.001^*^	3.17 (1.71~4.63)	<0.001^*^	3.10 (0.95~5.25)	0.005^*^
Gait speed	7.14 (5.52~8.76)	<0.001^*^	6.56 (4.85~8.27)	<0.001^*^	4.95 (3.52~6.38)	<0.001^*^	4.87 (3.43~6.31)	<0.001^*^

^a^Adjusted for sex, age, height, and smoking status. ^b^Adjusted for sex, height, and marital status. ^*^p < 0.05. Abbreviations: MM%: muscle mass percentage; AMM%: appendicular muscle mass percentage; ArmsMM%: arms muscle mass percentage; LegsMM%: legs muscle mass percentage; TrunkMM%: trunk muscle mass percentage; FM%: fat mass percentage; AFM%: appendicular fat mass percentage: ArmsFM%: arms fat mass percentage; LegsFM%: legs fat mass percentage; TrunkFM%: trunk fat mass percentage; FMR: fat-to-muscle ratio.

### Linear regression model of body compositions and muscle strength with QoL stratified by gender

In Table 4, we checked the factors associated with the SF-12 scores stratified by gender. After adjusting for age, height, and smoking status in males, PCS scores were positively associated with AMM% (β = 3.37; 95% confidence interval (CI) 0.27~6.47; *p* = 0.034), LegsMM% (β = 3.67; 95% CI 0.64~6.69; *p* = 0.018) and gait speed (β = 6.09; 95% CI 3.88~8.30; *p* < 0.001) and negatively associated with ArmsFM% (β = −5.00; 95% CI −9.27~−0.73; *p* = 0.023) and FMR (β = −4.31; 95% CI −8.08~−0.54; *p* = 0.026). In females, the results were similar to the overall results shown in Table 3, except that TrunkFM% (*p* = 0.090) had no significant association with PCS scores, and the strongest predictor of PCS was HGS (β = 10.54; 95% CI 6.27~14.81; *p* < 0.001). In addition, the interaction between gender and ArmsMM% as well as HGS was statistically significantly different (*p*-interaction = 0.032, 0.001 respectively). Briefly, the factors of PCS are gender dependent; gait speed plays a more weighing role in male while handgrip strength, in female.

**Table 4:** The correlation of body compositions and muscle strength with QoL stratified by gender in linear regression model.

	**PCS**			**MCS**		
	**Male**	**Female**	***p*-interaction^a^**	**Male**	**Female**	***p*-interaction^b^**
	**Standardized Adj-β (95% CI)^a^**	**Standardized Adj-β (95% CI)^a^**		**Standardized Adj-β (95% CI)^b^**	**Standardized Adj-β (95% CI)^b^**	
Body composition						
MM%	2.09 (−0.82~5.01)	3.35 (0.42~6.27)^*^	0.428	1.49 (−1.13~4.12)	2.64 (0.15~5.13)^*^	0.547
AMM%	3.37 (0.27~6.47)^*^	5.75 (2.45~9.05)^*^	0.203	2.32 (−0.45~5.08)	4.05 (1.26~6.83)^*^	0.362
ArmsMM%	1.75 (−1.31~4.82)	6.5 (2.34~10.66)^*^	0.032^*^	1.48 (−1.24~4.19)	3.2 (−0.34~6.74)	0.387
LegsMM%	3.67 (0.64~6.69)^*^	4.87 (1.92~7.81)^*^	0.459	2.43 (−0.29~5.15)	3.72 (1.24~6.21)^*^	0.481
TrunkMM%	0.58 (−2.12~3.27)	1.87 (−0.85~4.60)	0.458	0.49 (−1.97~2.94)	1.7 (−0.63~4.04)	0.524
FM%	−2.78 (−5.90~0.34)	−3.51 (−6.36~−0.65)^*^	0.675	−1.11 (−3.96~1.74)	−2.80 (−5.25~−0.36)^*^	0.394
AFM%	−4.48 (−9.02~0.06)	−3.88 (−6.84~−0.92)^*^	0.981	−1.88 (−6.01~2.24)	−2.19 (−4.74~0.36)	0.915
ArmsFM%	−5.00 (−9.27~−0.73)^*^	−3.01 (−5.73~−0.29)^*^	0.541	−2.08 (−5.99~1.83)	−3.40 (−5.72~−1.08)^*^	0.623
LegsFM%	−3.75 (−8.32~0.81)	−3.27 (−6.07~−0.47)^*^	0.984	−1.59 (−5.74~2.55)	−1.21 (−3.62~1.20)	0.883
TrunkFM%	−1.89 (−4.43~0.65)	−2.27 (−4.89~0.34)	0.803	−0.74 (−3.06~1.59)	−2.42 (−4.65~−0.19)^*^	0.320
FMR	−4.31 (−8.08~−0.54)^*^	3.95 (−6.55~−1.36)^*^	0.446	−2.56 (−5.98~0.86)	−3.03 (−5.25~−0.80)^*^	0.319
Muscle strength						
Handgrip strength	2.07 (−1.00~5.14)	10.54 (6.27~14.81)^*^	0.001^*^	0.42 (−2.18~3.02)	7.58 (4.00~11.17)^*^	0.001^*^
Gait speed	2.09 (−0.82~5.01)	7.1 (4.55~9.66)^*^	0.462	4.63 (2.66~6.60^)*^	5.14 (3.06~7.21)^*^	0.693

^a^Adjusted for age, height, and smoking status. ^b^Adjusted for height and marital status. ^*^p < 0.05. Abbreviations: MM%: muscle mass percentage; AMM%: appendicular muscle mass percentage; ArmsMM%: arms muscle mass percentage; LegsMM%: legs muscle mass percentage; TrunkMM%: trunk muscle mass percentage; FM%: fat mass percentage; AFM%: appendicular fat mass percentage; ArmsFM%: arms fat mass percentage; LegsFM%: legs fat mass percentage; TrunkFM%: trunk fat mass percentage; FMR: fat-to-muscle ratio.

After adjusting for height and marital status, in males, MCS scores were merely positively associated with gait speed (β = 4.63; 95% CI 2.66~6.60; *p* < 0.001). In females, the results were similar to the overall results shown in Table 3, and the strongest predictor of MCS was HGS (β = 7.58; 95% CI 4.00~11.17; *p* < 0.001), gait speed (β = 5.14; 95% CI 3.06~7.21; *p* < 0.001) came to the next. In addition, the interaction between gender and HGS was statistically significantly different (*p*-interaction = 0.001). Briefly, the factors of MCS are gender dependent; gait speed plays a more weighing role in male while handgrip strength, in female.

## DISCUSSION

This study demonstrated a gender difference in SF-12, body composition, and muscle strength in the elderly Taiwanese population. Gait speed plays a more weighing role in male while handgrip strength, in female. This implied that the muscle mass and strength of the lower limbs in men significantly affect their QoL. However, in women, ArmsMM% and HGS have the greatest impact on the improvement of SF-12 PCS, and HGS is also helpful for improving MCS, showing that the muscle mass and strength of upper limbs of female play a key role in improving the QoL.

Previous research have reported that lower muscle mass and higher fat mass are key factors to affect mobility and cause disability, thereby affecting QoL, consistent with our results [3, 19, 20, 34–36]. Furthermore, muscle strength, yet rapidly decline with aging and gender dependence, carries more contribution than muscle mass to QoL [37]. Another intriguing discovery is we found that PCS scores were positively associated with current or former smoking. This is probably because Taiwan’s law, Tobacco Hazards Prevention Act, has been stipulated that smoking is prohibited in all workplaces and public places since 2009, and it is necessary to move to outdoor designated smoking areas, which may increase the activity of smoking groups, thereby improving PCS scores. Reduction in muscle strength and mass will worsen QoL. Recent studies suggest that intervention of training program in high resistance and nutritional consultation based on individual needs are beneficial [20, 30]. In female, compared to gait speed, the handgrip strength shows higher β value in QoL, while gait speed, in male, implicating a differential intervention policy in promotion of QoL by gender difference. Therefore, our discovery may shed light on gender-dependent emphasis to precisely enhance QoL. Although we found gender differences in upper and lower limb muscle strength linking to QoL, further research is needed to elucidate mechanism behind biological significance.

Some studies propose no association between muscle mass and QoL, but we disclose the existence of association between them [38, 39]. Furthermore, the effect of muscle strength on QoL is more important than that of muscle mass, consistence with previous research, that is, muscle strength overwhelming muscle mass in determinants of QoL [39, 40]. If proper training in muscle strength is intervened, it would be conducive to QoL in older adults [9, 41].

This is a cross-sectional study, displaying the association between the body composition, muscle strength of upper and lower limbs and QoL. However, further cohort research is needed to elucidate cause and effect. The subjects of this study came from health examinations and they may have higher healthy literacy and cases source is needed to take into account in future research. Hence, these results may be applicable only to older adults who live in community-dwelling with relatively healthier status.

## CONCLUSIONS

An increase in muscle percentage and strength in elderly individuals will improve physical and psychological QoL, whereas an increase in fat percentage will decrease QoL. As there are gender differences in upper and lower limb muscle strength, men should focus on lower limb training, whereas women should focus on upper limb training to effectively improve QoL. Therefore, gender dependent exercise interventions should be considered in aging.
